# Electroactive PVDF-based composite thin films incorporating metal phosphate fillers as flexible dielectric substrates for dual-band microwave antennas

**DOI:** 10.1039/d6ra02658c

**Published:** 2026-08-12

**Authors:** Saïd Douhi, Chaymae Bahloul, Mounir El Achaby, Adil Eddiai

**Affiliations:** a Laboratory of Physics of Condensed Matter (LPMC), Faculty of Sciences Ben M'Sick, Hassan II University of Casablanca B.P. 7955 Sidi Othman Casablanca Morocco said.douhi.15@gmail.com adileddiai@gmail.com; b REMTEX Laboratory, Higher School of Textile and Clothing Industries (ESITH) Casablanca Morocco; c College of Chemical Sciences and Engineering (CCSE), Department of Materials Science, Energy and Nano-engineering (MSN), Mohammed VI Polytechnic University (UM6P) 43150 Benguerir Morocco

## Abstract

Flexible antennas represent key enabling components for emerging Internet-of-Things (IoT) platforms and next-generation wireless communication systems. This work presents a compact dual-band CPW-fed antenna based on electroactive PVDF composite thin films incorporating metal-phosphate fillers (Ni, Ag, and Co) as dielectric substrates. The proposed metamaterial-inspired configuration enables stable operation at 3.5 GHz and 5.8 GHz, covering the sub-6 GHz and ISM frequency bands relevant to modern wireless communication systems. Experimental validation performed on prototypes fabricated using PVDF/3Ni–P and PVDF/3Co–Pn substrates demonstrates good agreement with simulated results, confirming reliable dual-band performance. The antenna achieves measured impedance bandwidths of 3.17–3.50 GHz and 5.62–7.16 GHz, along with peak gains of 1.56 dBi and 5.32 dBi and radiation efficiencies reaching up to 91%. The results highlight that the developed PVDF-based composite substrates offer a favorable combination of mechanical flexibility, tunable dielectric properties, and stable electromagnetic performance under bending conditions. In addition, the SAR analysis confirms compliance with international safety limits, ensuring safe operation in proximity to the human body. Overall, this study demonstrates that electroactive PVDF composites incorporating metal-phosphate fillers can serve as effective multifunctional dielectric platforms for compact microwave antennas. These findings open new perspectives for the development of advanced flexible RF systems in 5G, Internet-of-Things (IoT), and wireless body area network (WBAN) applications.

## Abstract 2

Flexible antennas represent key enabling components for emerging Internet-of-Things (IoT) platforms and next-generation wireless communication systems.

## Graphical abstract

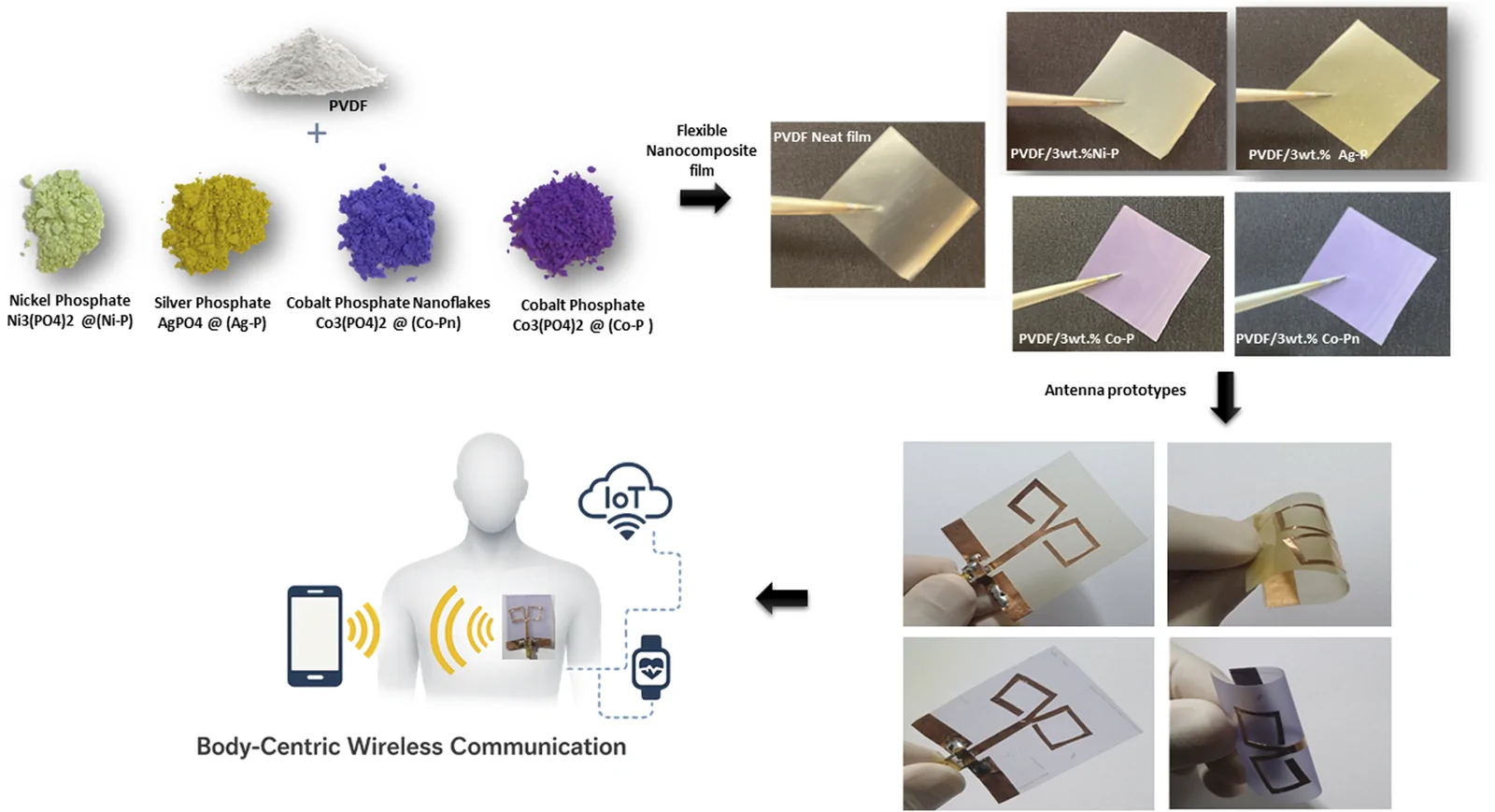

## 1 Introduction

In recent years, the rapid advancement of wireless communication technologies and wearable electronics has driven significant interest in flexible radio-frequency (RF) systems. These technologies enable the development of compact, body-centric devices capable of continuous data exchange and real-time monitoring. Consequently, flexible RF components are increasingly employed in a wide range of applications, including healthcare monitoring, security systems, wireless sensing, and Internet-of-Things (IoT) platforms.^1–7^ In parallel, a transition from conventional rigid substrates (*e.g.*, FR4 and RT/Duroid) to flexible electronic platforms is underway. Soft polymers such as polydimethylsiloxane (PDMS) and polyurethane (PU) have attracted considerable attention due to their mechanical compliance and ease of fabrication.^8^ This evolution aims to reduce system weight and cost while introducing additional degrees of freedom through flexibility, enabling conformability and mechanical adaptability. These developments support the realization of fully flexible and biocompatible systems, including wearable and implantable medical devices, thereby reinforcing the need for RF components—particularly antennas—capable of operating under mechanical deformation and body-loading conditions.^9^ Within this context, wearable and flexible antennas play a critical role in ensuring reliable RF signal transmission and reception. As key electromagnetic components, antennas enable efficient radiation and reception of radio waves while meeting the requirements of compactness and seamless integration into garments or directly onto the human body.^10^

The development of flexible antennas is therefore essential for next-generation wireless body area networks (WBANs), which offer promising solutions for personalized healthcare and medical diagnostics.^11,12^ Research efforts have focused on diverse design architectures, fabrication strategies, and advanced substrate materials to achieve conformal and low-profile operation. However, substrate material selection remains a critical challenge, as wearable antennas must simultaneously exhibit mechanical flexibility, stable dielectric properties, and robustness under body-loading conditions.^13^ These requirements motivate the investigation of functional polymer composites as promising substrates for next-generation wearable communication devices. Flexible substrates have emerged as an important solution for modern electronic devices, offering enhanced design flexibility while minimizing the total dimensions and mass of electronic systems. For this reason, flexible electronic technologies have received significant research attention in recent decades from both academic and industrial communities.^14^ Polymers are widely used as substrates for flexible antennas,^15–17^ with polyimide,^18,19^ polytetrafluoroethylene (PTFE),^20^ polyethylene terephthalate (PET),^21^ and liquid crystal polymer (LCP)^22^ demonstrating varying suitability for RF applications.

Piezoelectric materials are widely employed in advanced technologies such as energy harvesting systems, transducers, actuators, sensors, and microwave antennas owing to their unique electromechanical coupling properties. In recent years, poly(vinylidene fluoride) (PVDF)-based piezoelectric materials have attracted considerable attention for multifunctional wireless electronics. They have been successfully integrated into dual-band coplanar waveguide antennas operating at 2.45/5.8 GHz for RF energy harvesting, hybrid RF–vibration energy harvesting antennas, and compact metasurface absorbers capable of simultaneously harvesting ambient radio-frequency and vibrational energy.^23–25^ These studies highlight the remarkable versatility of PVDF in enabling the simultaneous conversion of electromagnetic and mechanical energy within lightweight, flexible, and multifunctional platforms. Nevertheless, despite these significant advances, the development of next-generation piezoelectric materials exhibiting simultaneously high dielectric permittivity, low dielectric loss, enhanced piezoelectric response, and excellent mechanical flexibility remains a major scientific challenge.^26,27^ To meet these requirements, PVDF and its copolymers have been extensively investigated owing to their excellent chemical stability, mechanical flexibility, biocompatibility, ease of processing, and outstanding electroactive properties.^28–34^ However, the intrinsic dielectric and piezoelectric performances of pristine PVDF are still insufficient for many high-performance microwave and energy harvesting applications. Consequently, considerable research efforts have focused on enhancing the functional properties of PVDF through the incorporation of inorganic fillers. Among the various candidates, metal phosphate-based particles (MP–Ps) have emerged as particularly attractive fillers because of their high chemical stability, low cost, environmental friendliness, and strong interfacial interactions with PVDF molecular chains. Their incorporation can effectively enhance dielectric polarization, improve interfacial compatibility, reinforce the mechanical properties, and promote the formation of the electroactive β-phase, thereby improving the overall electromechanical performance of the composite. Furthermore, recent advances in materials engineering have demonstrated that the morphology, particle size, crystallinity, and surface chemistry of MP–Ps can be precisely tailored through synthesis techniques such as ball milling, chemical precipitation, and hydrothermal processing, enabling fine control over the microstructure and functional properties of the resulting composites.^35,36^ Consequently, PVDF/MP–P composites have emerged as promising multifunctional materials for flexible microwave devices, wearable antennas, RF energy harvesting systems, and next-generation wireless communication technologies.

In this work, a compact coplanar waveguide (CPW)-fed dual-band antenna based on a metamaterial-inspired configuration is developed using electroactive PVDF composite thin films incorporating metal phosphate fillers (Ni, Ag, and Co) as flexible dielectric substrates. The proposed antenna operates in the sub-6 GHz range, covering the 3.5 GHz WiMAX/5G band and the 5.8 GHz industrial, scientific, and medical (ISM) band for IoT applications. The dielectric properties of the developed composites are experimentally characterized, and prototype antennas are fabricated and validated in terms of impedance matching, gain, and radiation efficiency, showing good agreement with simulations. Furthermore, the antenna performance under mechanical deformation is investigated, and its specific absorption rate (SAR) is evaluated to ensure safe on-body operation. These results demonstrate the potential of PVDF-based electroactive composites as multifunctional substrates for flexible antennas in 5G, IoT, and wireless body area networks (WBAN).

## 2 Materials and antenna design

### 2.1 Fabrication of PVDF-based composite thin films

**Fig. 1:**
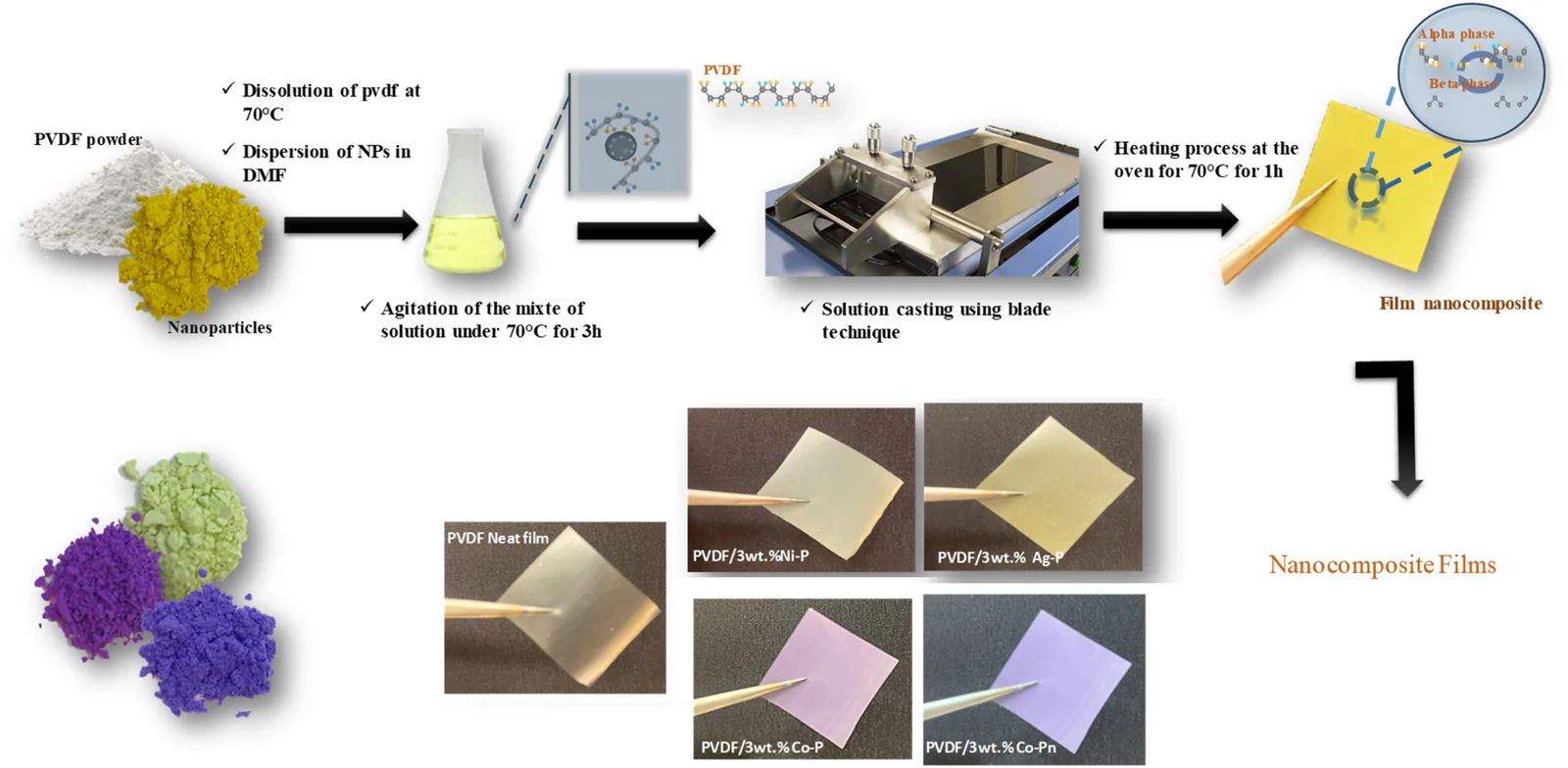
Schematic illustration of the fabrication process of electroactive PVDF-based composite thin films incorporating metal phosphate fillers.

**Fig. 2:**
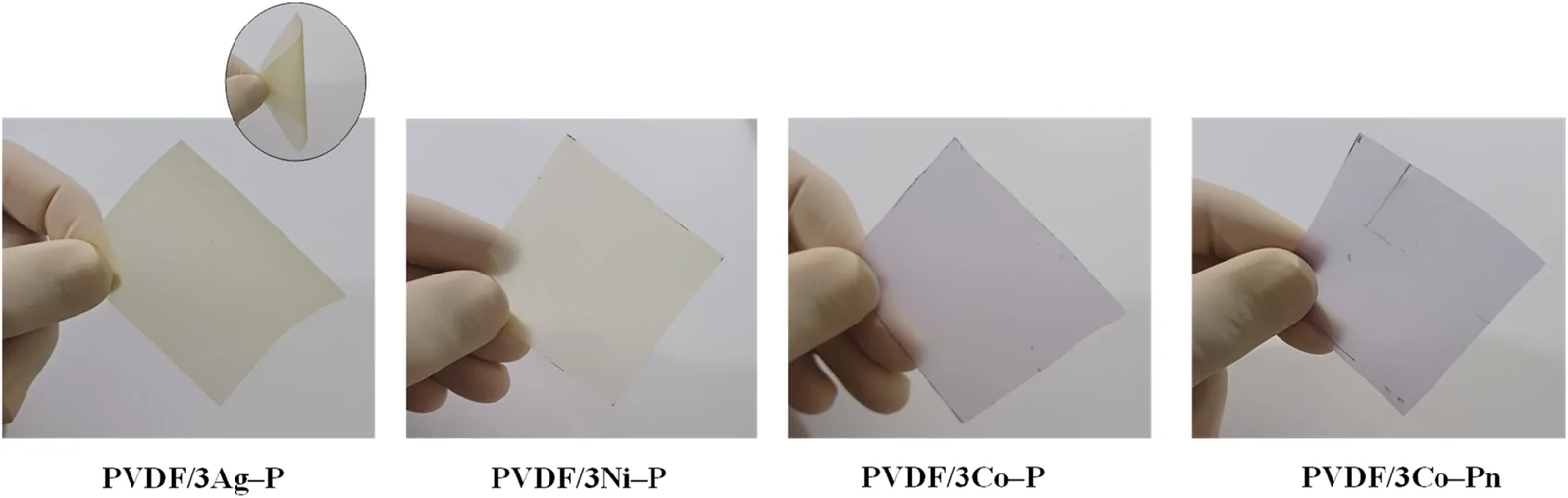
Photographs of the fabricated flexible PVDF-based composite thin films: PVDF/3Ag–P, PVDF/3Ni–P, PVDF/3Co–P, and PVDF/3Co–Pn.

The PVDF-based composite thin films employed in this study were previously developed and comprehensively characterized in our earlier work.^37^ In the present study, these materials are utilized as flexible dielectric substrates for antenna applications. For completeness, a concise description of the fabrication procedure is provided. Poly(vinylidene fluoride) (PVDF) was used as the polymer matrix due to its excellent flexibility, chemical stability, and favorable functional properties. Metal phosphate-based particles (MP-Ps), including Ni–P, Ag–P, Co–P, and Co–Pn, were incorporated as fillers to tailor the composite properties. Based on the optimization results reported in our previous study, a filler concentration of 3 wt% was selected, as it provides a good balance between homogeneous dispersion, mechanical flexibility, and preservation of the electroactive β-phase. The composite films were prepared *via* a solution casting approach. PVDF powder was first dissolved in dimethylformamide (DMF) under continuous stirring at an elevated temperature (approximately 70 °C) until a clear and homogeneous solution was obtained. Separately, the required amount of MP-Ps (3 wt%) was dispersed in DMF using ultrasonic agitation to ensure uniform distribution of the particles. The particle dispersion was then gradually introduced into the PVDF solution under continuous stirring, followed by additional ultrasonication to enhance the homogeneity of the mixture. The resulting solution was subsequently cast onto a clean glass substrate using a doctor blade technique to control the film thickness. After casting, the films were dried at moderate temperature to allow complete solvent evaporation. The dried films were then carefully peeled off, resulting in flexible and uniform composite thin films. The fabricated samples are denoted as PVDF/3Ni–P, PVDF/3Ag–P, PVDF/3Co–P, and PVDF/3Co–Pn, depending on the filler type, while a pure PVDF film was also prepared for comparison. These composite films exhibit good flexibility, uniform morphology, and structural integrity, making them suitable candidates for subsequent integration as dielectric substrates in antenna systems. Fig. 1 illustrates the schematic fabrication process of the electroactive PVDF-based composite thin films incorporating metal phosphate fillers, while Fig. 2 presents photographs of the fabricated flexible samples, including (a) PVDF/7Ag–P, (b) PVDF/7Ni–P, (c) PVDF/3Co–P, and (d) PVDF/3Co–Pn.

### 2.2 Dielectric characterization

**Fig. 3:**
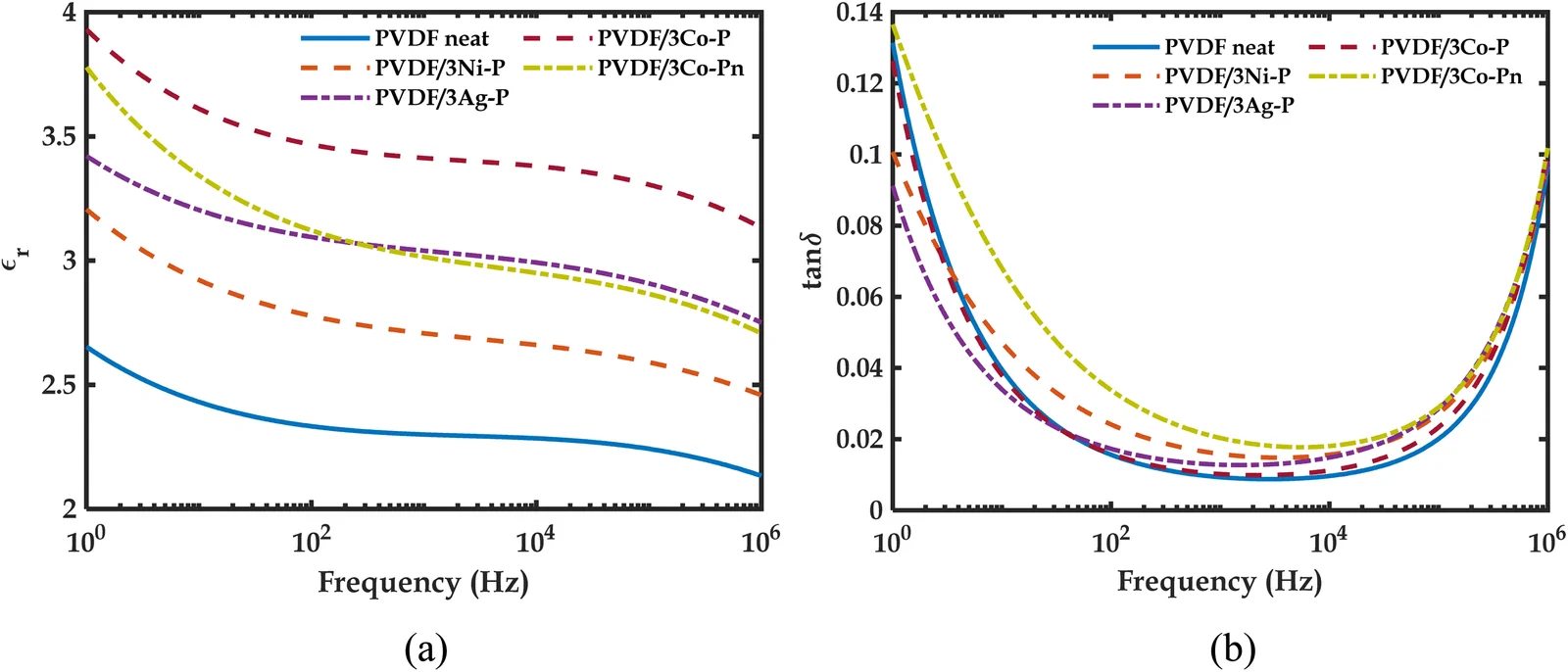
Frequency dependence of (a) relative permittivity (*ε*_r_) and (b) dielectric loss tangent (tan *δ*) for the investigated PVDF-based composite substrates.

The dielectric properties of the fabricated PVDF-based composite thin films were investigated to evaluate their suitability as flexible substrates for microwave antenna applications. The effective relative permittivity (*ε*_r_) and dielectric loss tangent (tan *δ*) of neat PVDF and PVDF-based composite films containing 3 wt% of different metal phosphate fillers (Ni–P, Ag–P, Co–P, and Co–Pn) were measured over the frequency range from 1 Hz to 1 MHz. It should be emphasized that the dielectric measurements were performed on the final composite films rather than on the isolated filler particles, since the effective dielectric properties of the composite substrates are the parameters directly required for electromagnetic antenna design. Fig. 3(a) presents the variation of the relative permittivity as a function of frequency for all investigated samples. Compared with neat PVDF, all composite formulations exhibit an increase in dielectric permittivity over the entire investigated frequency range, confirming the beneficial effect of incorporating metal phosphate fillers into the PVDF matrix. This enhancement is mainly attributed to the combined effect of interfacial polarization at the filler–polymer interfaces, commonly described by the Maxwell–Wagner–Sillars (MWS) mechanism, together with the increased dipolar polarization associated with the enhanced electroactive β-phase content of PVDF. The dielectric response also depends on the nature of the incorporated filler. Among the investigated formulations, PVDF/3Co–P exhibits the highest dielectric constant, while PVDF/3Co–Pn also presents significantly enhanced permittivity owing to the strong interfacial interactions and favorable particle morphology achieved through the different synthesis routes. The PVDF/3Ag–P and PVDF/3Ni–P composites also show improved dielectric properties compared with neat PVDF, demonstrating that all investigated metal phosphate fillers effectively contribute to the enhancement of the dielectric response. From a practical perspective, improving the dielectric properties of polymer composites through inorganic filler incorporation may influence their mechanical flexibility. However, by optimizing the filler loading to 3 wt%, the developed PVDF-based composites achieve a suitable compromise between dielectric enhancement and mechanical compliance. As demonstrated in our previous comprehensive mechanical characterization of the same composite systems,^38^ the optimized formulations retained excellent flexibility while exhibiting improved mechanical properties. This balance is particularly important for flexible antenna substrates, where favorable dielectric characteristics must be achieved without sacrificing the mechanical flexibility required for wearable and conformal applications. As shown in Fig. 3(b), the dielectric loss tangent exhibits a rapid decrease with increasing frequency in the low-frequency region, followed by a relatively stable behavior and a slight increase at higher frequencies. This frequency dependence is characteristic of dipolar and interfacial relaxation processes. The relatively high losses observed at very low frequencies are mainly associated with charge accumulation at the filler–polymer interfaces through the Maxwell–Wagner–Sillars polarization mechanism. As the frequency increases, the dipoles become progressively unable to follow the alternating electric field, resulting in reduced dielectric losses. Although the incorporation of metal phosphate fillers slightly increases the dielectric loss compared with neat PVDF, all investigated composites maintain moderate tan *δ* values over most of the investigated frequency range. Such dielectric losses remain sufficiently low for microwave substrate applications, ensuring that the enhancement in dielectric permittivity is achieved without significantly compromising the electromagnetic performance of the antenna. It is important to note that the dielectric properties reported here correspond to low-frequency measurements (1 Hz to 1 MHz). Although these measurements provide valuable insight into the polarization mechanisms governing the dielectric behavior of the developed composites, a similar methodology has been successfully adopted in previous studies,^38^ where low-frequency dielectric characterization was employed to develop microwave antenna substrates, and the suitability of the materials was ultimately validated through antenna measurements. In the present work, the applicability of the proposed PVDF-based composite substrates for microwave antenna applications is therefore further confirmed by the excellent agreement obtained between the simulated and measured antenna characteristics. Overall, the developed PVDF-based composite films exhibit enhanced dielectric properties while maintaining moderate dielectric losses and good mechanical flexibility. These characteristics demonstrate that the proposed electroactive PVDF composites constitute promising flexible dielectric substrates for dual-band microwave antennas intended for wearable, IoT, and wireless communication applications.

### 2.3 Antenna design and performance analysis

**Fig. 4:**
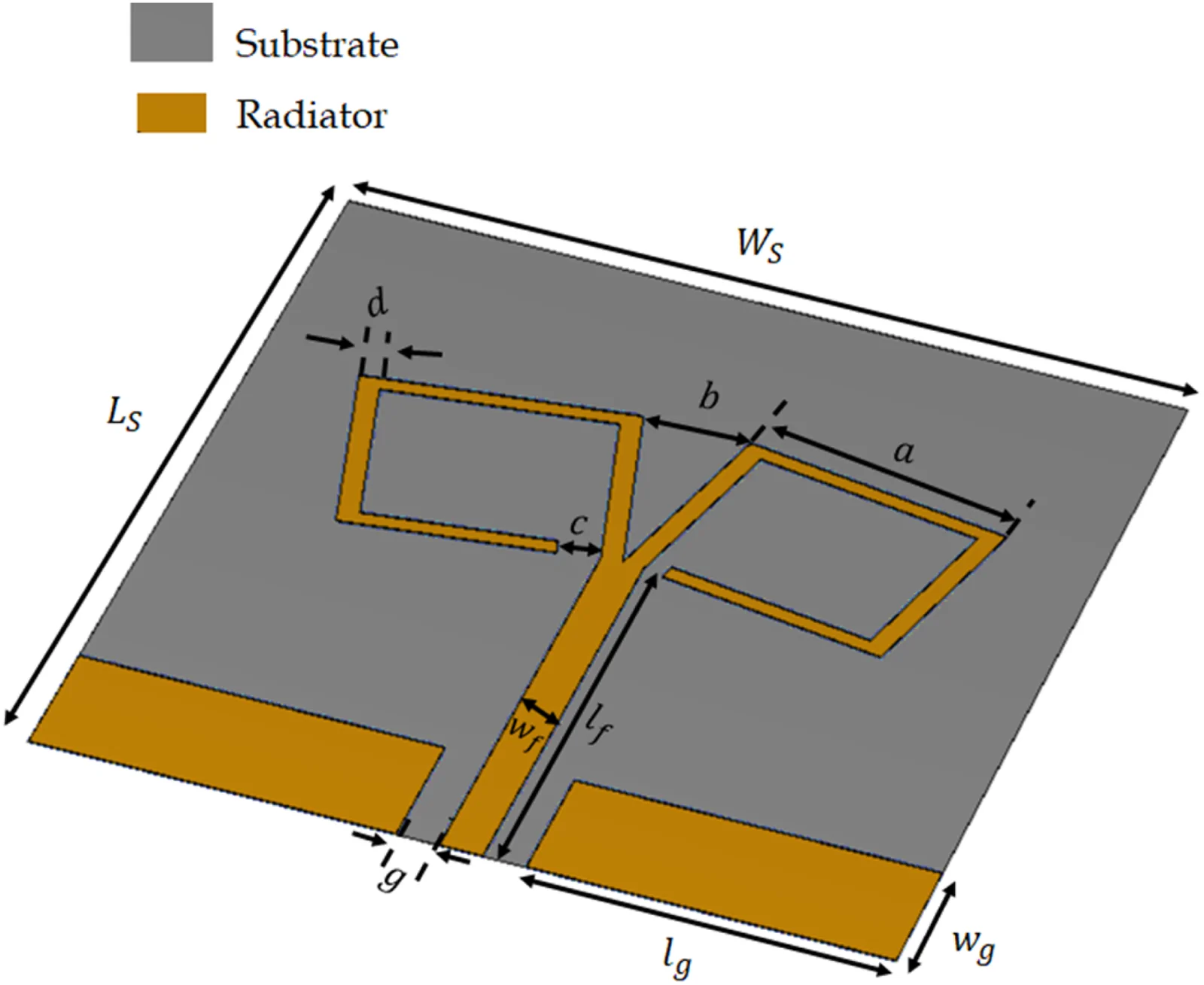
Geometry and configuration of the proposed CPW-fed dual-band antenna implemented on PVDF-based composite substrate.

Planar antennas based on microstrip technology are widely employed in modern wireless communication systems due to their compact size, low profile, and ease of fabrication. Their compatibility with standard printed circuit technologies enables straightforward integration into various platforms, including portable devices, automotive systems, and wearable electronics. In particular, their planar geometry and reduced thickness make them highly suitable for flexible and conformal applications. The proposed antenna configuration is illustrated in Fig. 4. The design adopts a coplanar waveguide (CPW)-fed structure implemented on a flexible PVDF-based composite thin-film substrate incorporating metal-phosphate fillers (M = Ni, Ag, Co) with a fixed concentration of 3 wt%. This composite substrate provides both mechanical flexibility and enhanced dielectric characteristics, making it a suitable candidate for wearable antenna applications. The antenna occupies a compact footprint of 40 × 52 mm^2^, facilitating its integration into wearable communication systems. The radiating element consists of two symmetrically arranged square-ring resonators connected to a central feed line. The antenna is excited through a CPW feed of width *W*_f_ and length *L*_f_, separated from the ground planes by a gap *g*. The ground planes, defined by dimensions *l*_g_ × *w*_g_, ensure stable impedance matching and efficient power transfer. Each square-ring resonator is characterized by its outer side length *a*, the spacing *b* between the upper arms, and the internal offset *c*, while the strip width is denoted by *d*. This configuration enables dual-band operation at approximately 3.5 GHz and 5.8 GHz, corresponding to sub-6 GHz 5G/WiMAX and WLAN/ISM bands, respectively. Compared with conventional rigid substrates such as FR-4,^39^ the proposed PVDF-based composite substrates offer enhanced electroactive polarization, tunable dielectric properties, and superior mechanical flexibility. These characteristics enable improved conformability to curved surfaces, such as the human body, while maintaining stable electromagnetic performance. The optimized geometrical parameters of the antenna are summarized in Table 1. The electromagnetic performance of the proposed design was analyzed using full-wave simulations carried out in CST Microwave Studio.

**Table 1:** Optimized dimensions of the suggested antenna

Parameters	Value (mm)
*W*_s_	40
*L*_s_	52
*L*_f_	26.9
*W*_f_	2.0
*l*_g_	8
*w*_g_	17
*g*	2
*a*	13
*b*	5.07
*c*	2
*d*	1

**Fig. 5:**
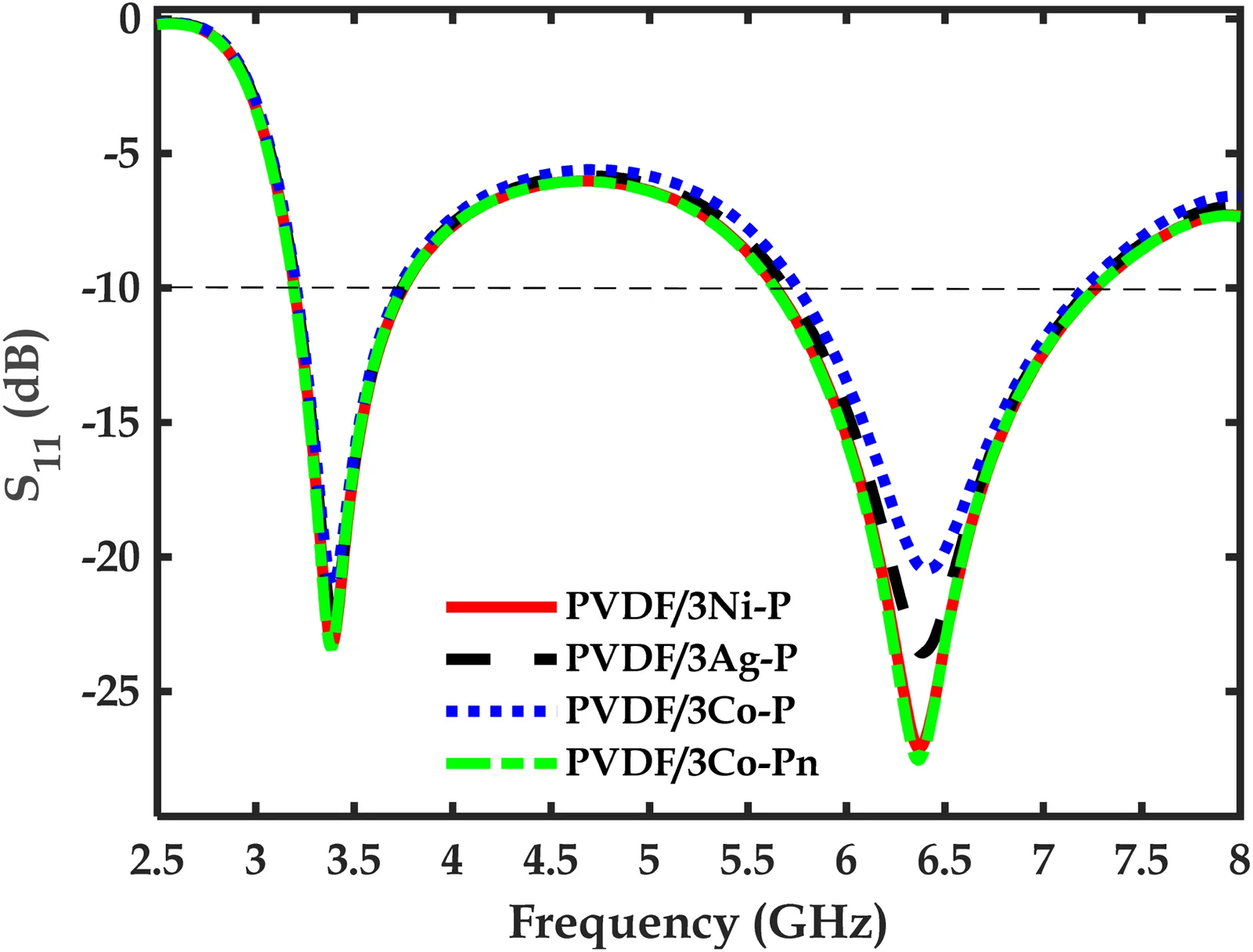
Simulated reflection coefficient (|*S*_11_|) of the proposed antenna for different PVDF-based composite substrates incorporating metal-phosphate fillers.

**Fig. 6:**
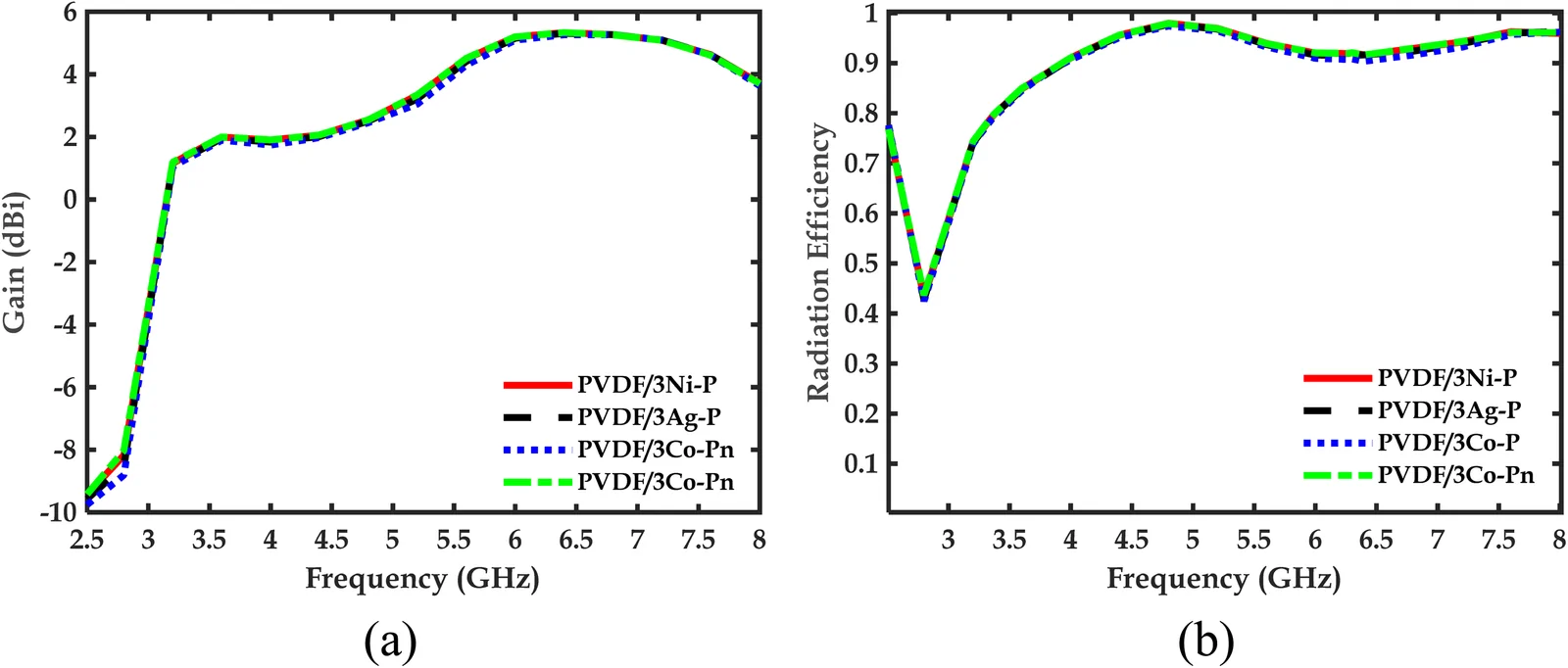
Simulated radiation performance of the proposed antenna: (a) gain and (b) radiation efficiency for different PVDF-based composite substrates.

**Fig. 7:**
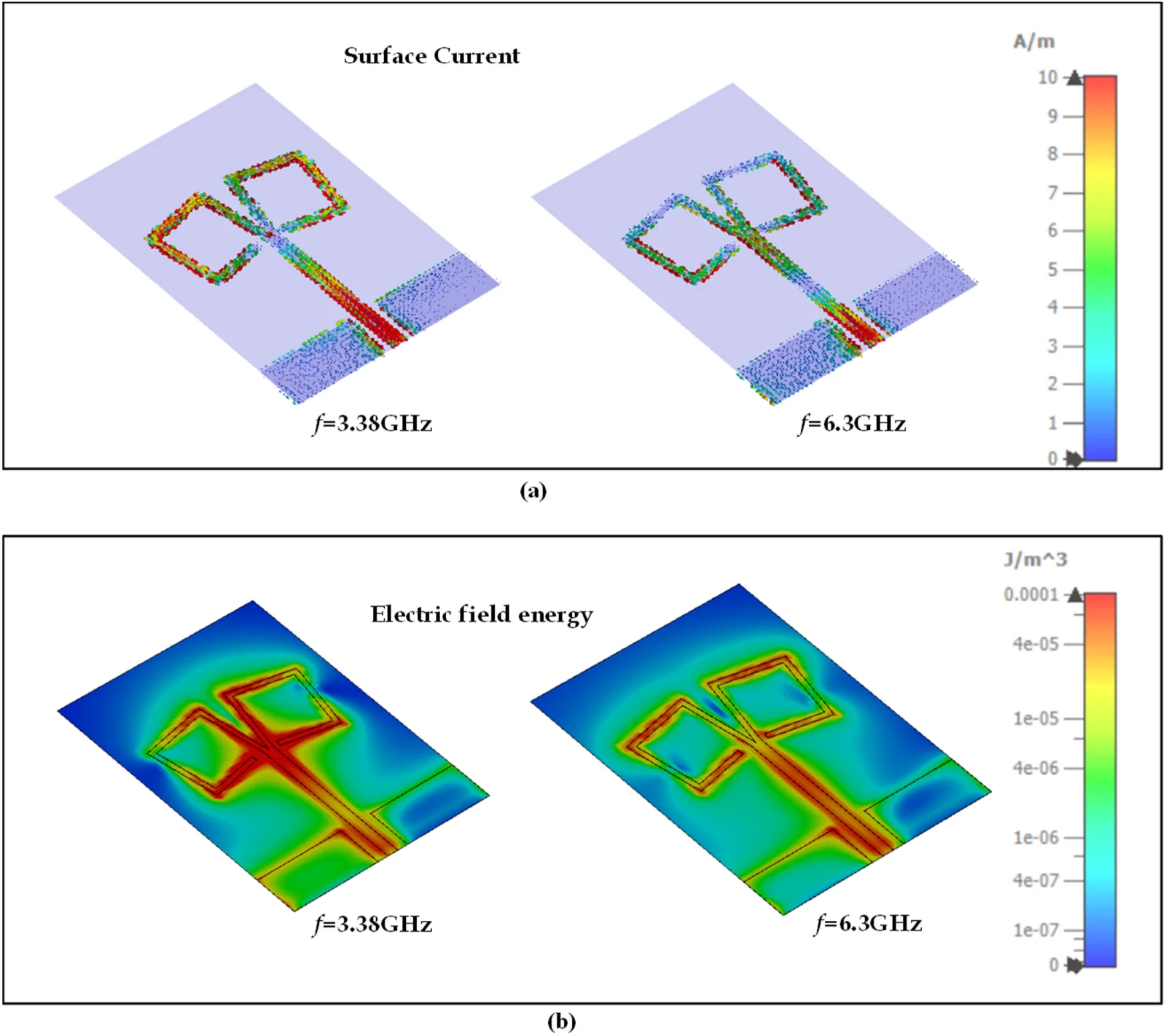
Simulated field distributions of the proposed antenna: (a) surface current distribution and (b) electric field distribution at the resonant frequencies.

The simulated reflection coefficient (∣*S*_11_∣) of the proposed antenna for different PVDF-based composite substrates incorporating metal-phosphate fillers at 3 wt% is presented in Fig. 5. The results demonstrate that the antenna maintains stable dual-band operation across all investigated substrates, with only minor variations in resonance depth and slight frequency shifts. Two distinct resonant modes are observed around 3.39 GHz and 6.3–6.4 GHz, corresponding to the targeted sub-6 GHz 5G and ISM bands, respectively. All composite substrates exhibit good impedance matching, with ∣*S*_11_∣ values below −20 dB in both frequency bands. The observed variations in the reflection coefficient can be attributed to differences in the effective permittivity and dielectric losses of the composite substrates. Materials exhibiting slightly higher effective permittivity lead to a shift in the resonant frequency, while the level of dielectric losses influences the depth of the resonance. In particular, substrates with an optimized balance between permittivity and loss tangent provide deeper resonance levels and improved impedance matching, indicating more efficient power transfer from the feed line to the radiating structure. The corresponding simulated radiation characteristics, including gain and radiation efficiency, are presented in Fig. 6. Both parameters show consistent behavior across the operating bands, confirming stable radiation performance for all investigated composites. The simulated gain varies from 1.45 to 1.56 dB in the lower band and reaches up to 5.32 dB in the upper band, while the radiation efficiency ranges between 79% and 91%. These variations are mainly governed by the dielectric properties of the substrates, where lower dielectric losses contribute to higher radiation efficiency and improved gain. It is observed that certain composite substrates provide a more favorable electromagnetic response, combining deeper impedance matching with slightly enhanced gain and efficiency. This behavior indicates a more efficient excitation of the resonant modes and reduced dissipative losses within the substrate. Such characteristics are essential for achieving reliable antenna performance in practical applications. Based on these results, the PVDF/3Co–Pn and PVDF/3Ni–P composite substrates demonstrate the most balanced performance, offering improved impedance matching along with higher gain and radiation efficiency. This can be attributed to their optimized dielectric properties, which ensure an effective trade-off between polarization enhancement and dielectric losses. Consequently, these substrates are selected for further experimental validation. Fig. 7 illustrates the simulated surface current and electric field distributions of the proposed antenna at the two resonant frequencies. At 3.39 GHz, the surface current is mainly concentrated along the CPW feed line and the inner edges of the square-ring resonators, corresponding to the fundamental resonant mode. At 6.3 GHz, the current distribution shifts toward the outer edges of the resonators, indicating the excitation of a higher-order resonant mode. In both cases, the electric field is strongly confined around the resonant structure and the feeding region, confirming efficient electromagnetic coupling and validating the dual-band operation of the antenna.

## 3 Results and discussion

### 3.1 Reflection coefficient (*S*_11_) analysis

**Fig. 8:**
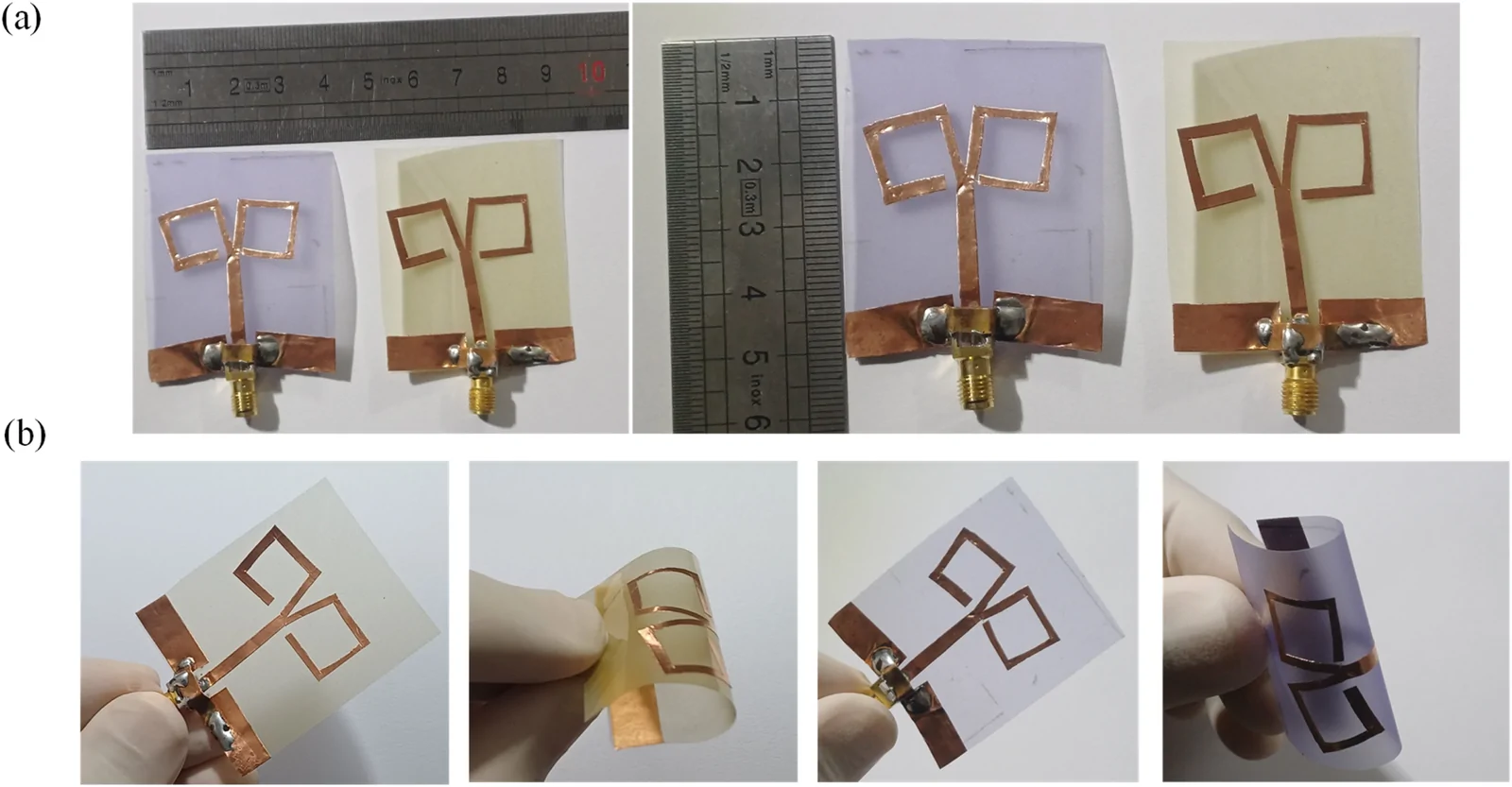
Fabricated dual-band antenna prototype: (a) dimensional verification of the antenna geometry; (b) demonstration of its mechanical flexibility.

**Fig. 9:**
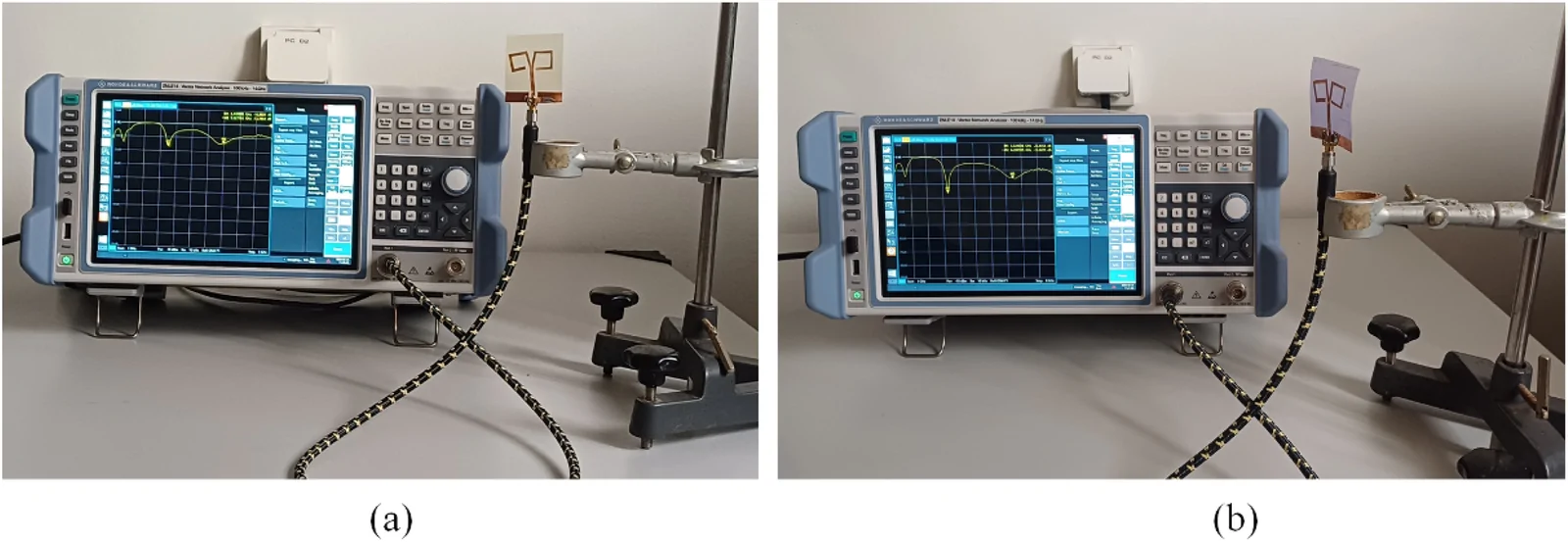
Fabricated antenna prototypes based on PVDF composite substrates: (a) PVDF/3Ni–P; (b) PVDF/3Co–Pn.

**Fig. 10:**
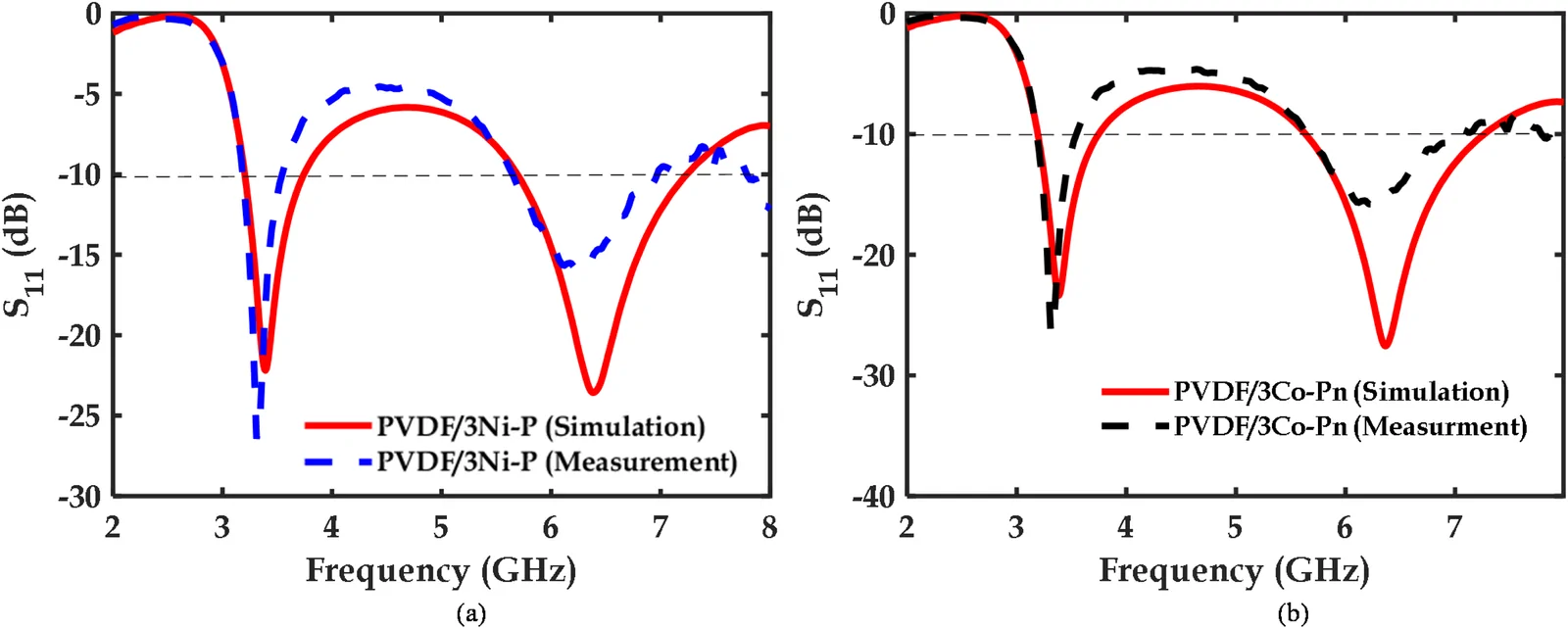
Simulated and measured reflection coefficient (|*S*_11_|) of the proposed antenna under free-space conditions: (a) PVDF/3Ni–P; (b) PVDF/3Co–Pn substrate.

To experimentally validate the proposed antenna design, prototype antennas were fabricated and characterized using a vector network analyzer (Rohde & Schwarz ZNLE14). The antenna was connected *via* a standard SMA connector, and the reflection coefficient (|*S*_11_|) was measured to evaluate the impedance matching performance. Photographs of the fabricated antenna are shown in Fig. 8. Fig. 8(a) confirms the dimensional accuracy of the realized structure, while Fig. 8(b) demonstrates its mechanical flexibility, highlighting its capability for conformal applications. Prior to prototype fabrication, the proposed antenna was numerically investigated using the measured dielectric properties of all fabricated PVDF-based composite substrates. As summarized in Table 2, the four investigated formulations exhibited comparable electromagnetic performance, with only minor differences in impedance matching, operating bandwidth, realized gain, and radiation efficiency. Since the objective of this study was to demonstrate the feasibility of electroactive PVDF composite substrates as flexible dielectric materials for microwave antenna applications, fabricating and experimentally characterizing all four antenna configurations would have provided only limited additional insight. Therefore, two representative composite substrates, namely PVDF/3Ni–P and PVDF/3Co–Pn, were selected for prototype fabrication and experimental validation. These two formulations exhibited favorable dielectric characteristics together with excellent simulated antenna performance, making them suitable representative candidates for validating the proposed concept. The fabricated antenna prototypes implemented on the selected PVDF-based composite substrates are presented in Fig. 9, including PVDF/3Ni–P and PVDF/3Co–Pn. The measured reflection coefficients of the proposed antenna are depicted in Fig. 10. Both configurations clearly exhibit dual-band resonance behavior, in good agreement with the simulated results presented in Section 2.3. Minor frequency shifts are observed between simulated and measured results, which can be attributed to fabrication tolerances, connector effects, and slight variations in the dielectric properties of the composite substrates. For the PVDF/3Ni–P substrate, the first resonance occurs at 3.31 GHz with a −10 dB impedance bandwidth ranging from 3.17 to 3.50 GHz. The second resonance is observed at 6.14 GHz, with a bandwidth extending from 5.62 to 6.90 GHz. For the PVDF/3Co–Pn substrate, a similar lower-frequency resonance is obtained, while the upper resonance shifts slightly to 6.20 GHz and exhibits a wider bandwidth of 5.62–7.16 GHz, thereby improving coverage of the upper operating band. These results indicate that the lower resonant frequency is primarily governed by the antenna geometry, whereas the upper resonance is more sensitive to the effective dielectric properties of the substrate. In particular, variations in relative permittivity influence the resonant frequency position, while dielectric losses affect the resonance depth and impedance matching characteristics. The slight differences observed between the two fabricated substrates are consistent with their measured dielectric properties and further demonstrate the influence of the composite dielectric material on the antenna response. Overall, the measured results are in excellent agreement with the numerical predictions, confirming the accuracy of the electromagnetic model and the reproducibility of the proposed fabrication process. The good agreement between simulation and experiment validates the suitability of the developed electroactive PVDF composite films as flexible dielectric substrates for compact dual-band microwave antennas operating within the targeted sub-6 GHz frequency bands.

**Table 2:** Simulated performance of the suggested dual-band antenna using different PVDF composite dielectric substrates loaded with metal phosphate-based particles

PVDF composite substrate	|*S*_11_| (dB)	Resonant frequencies & bandwidth (GHz)	Peak gain (dB)	Radiation efficiency (%)
PVDF/3Ni–P	−23.25/−27.08	3.38 (3.19–3.74)/6.38 (5.64–7.25)	1.45/5.23	80/91
PVDF/3Ag–P	−22.18/−23.52	3.39 (3.20–3.70)/6.40 (5.60–7.23)	1.51/5.31	80/91
PVDF/3Co–P	−21.05/−20.45	3.39 (3.20–3.70)/6.41 (5.75–7.20)	1.45/5.26	79/90
PVDF/3Co–Pn	−22.97/−27.39	3.39 (3.19–3.47)/6.30 (5.46–7.26)	1.56/5.32	80/91

### 3.2 Bending effect analysis

**Fig. 11:**
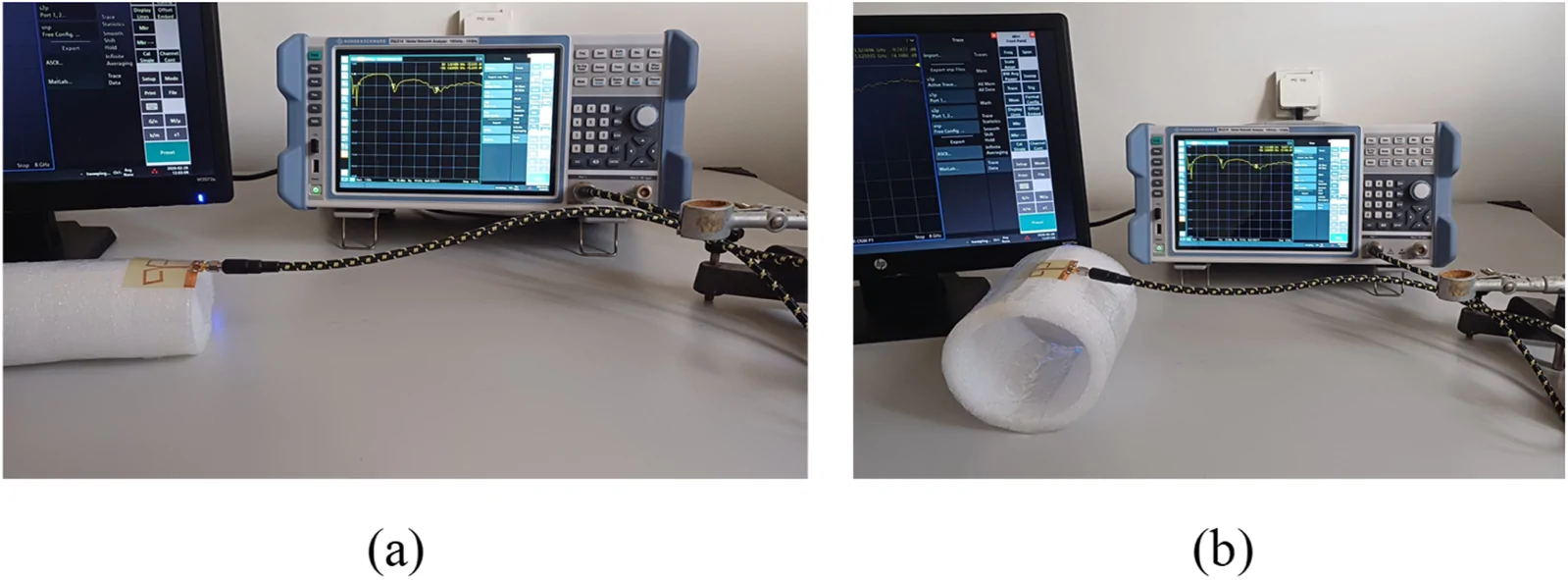
Experimental setup for mechanical flexibility testing: the antenna mounted on a cylindrical support to induce bending along (a) the *x*-direction and (b) the *y*-direction.

**Fig. 12:**
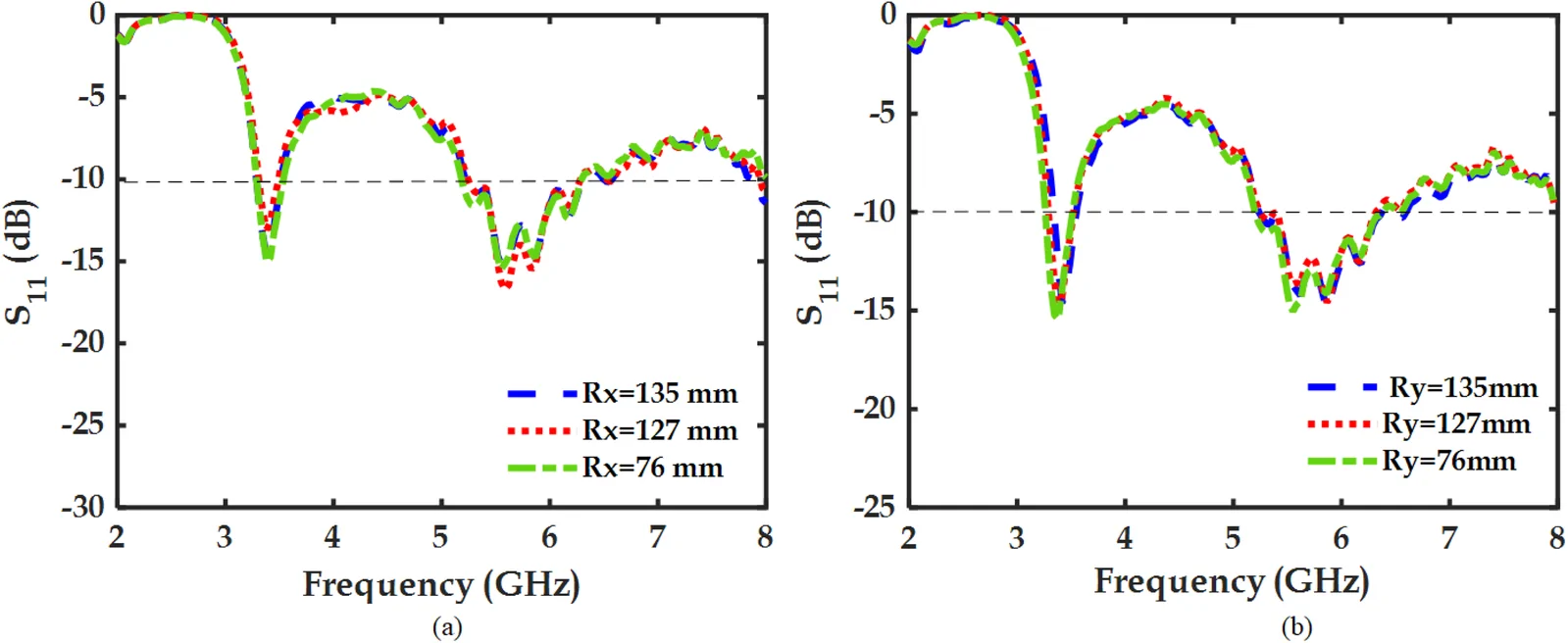
Measured reflection coefficient (|*S*_11_|) of the proposed antenna under various bending conditions: (a) *x*-direction and (b) *y*-direction.

In wearable antenna systems, mechanical deformation caused by body movement and the irregular geometry of human surfaces is unavoidable during practical operation. Therefore, evaluating the stability of antenna performance under bending conditions is essential. To investigate this aspect, bending experiments were performed on the fabricated antenna implemented on the PVDF/3Ni–P composite substrate. The measurements were conducted under free-space conditions by conformally attaching the antenna to cylindrical polystyrene supports with diameters of 76 mm, 127 mm, and 135 mm, corresponding approximately to the curvature of the human arm, thigh, and chest, respectively. During the measurements, the antenna was fixed using adhesive tape to ensure stable positioning. Bending tests were applied along both the *x*- and *y*-directions, as illustrated in Fig. 11(a) and (b), enabling the evaluation of electromagnetic performance under different deformation orientations. The corresponding reflection coefficient (|*S*_11_|) for various bending radii is presented in Fig. 12. The results indicate that the antenna maintains stable dual-band operation under all bending conditions. The lower resonant mode remains nearly unchanged, with only minor variations in impedance matching, indicating that this resonance is primarily governed by the antenna geometry and is weakly affected by moderate deformation. In all cases, the reflection coefficient remains below −10 dB, confirming stable impedance characteristics. In contrast, the higher-frequency resonance exhibits slightly greater sensitivity to bending, leading to small frequency shifts and variations in resonance depth under tighter curvature. This behavior can be attributed to changes in the effective electrical path length, surface current distribution, and slight variations in the effective dielectric properties of the composite substrate under deformation. Similar responses are observed for bending along both principal directions, indicating good mechanical isotropy and structural robustness of the antenna. Overall, these results demonstrate that the proposed PVDF-based composite antenna exhibits excellent mechanical flexibility while maintaining stable electromagnetic performance, making it well suited for conformal and wearable wireless communication applications.

### 3.3 On-body performance analysis

**Fig. 13:**
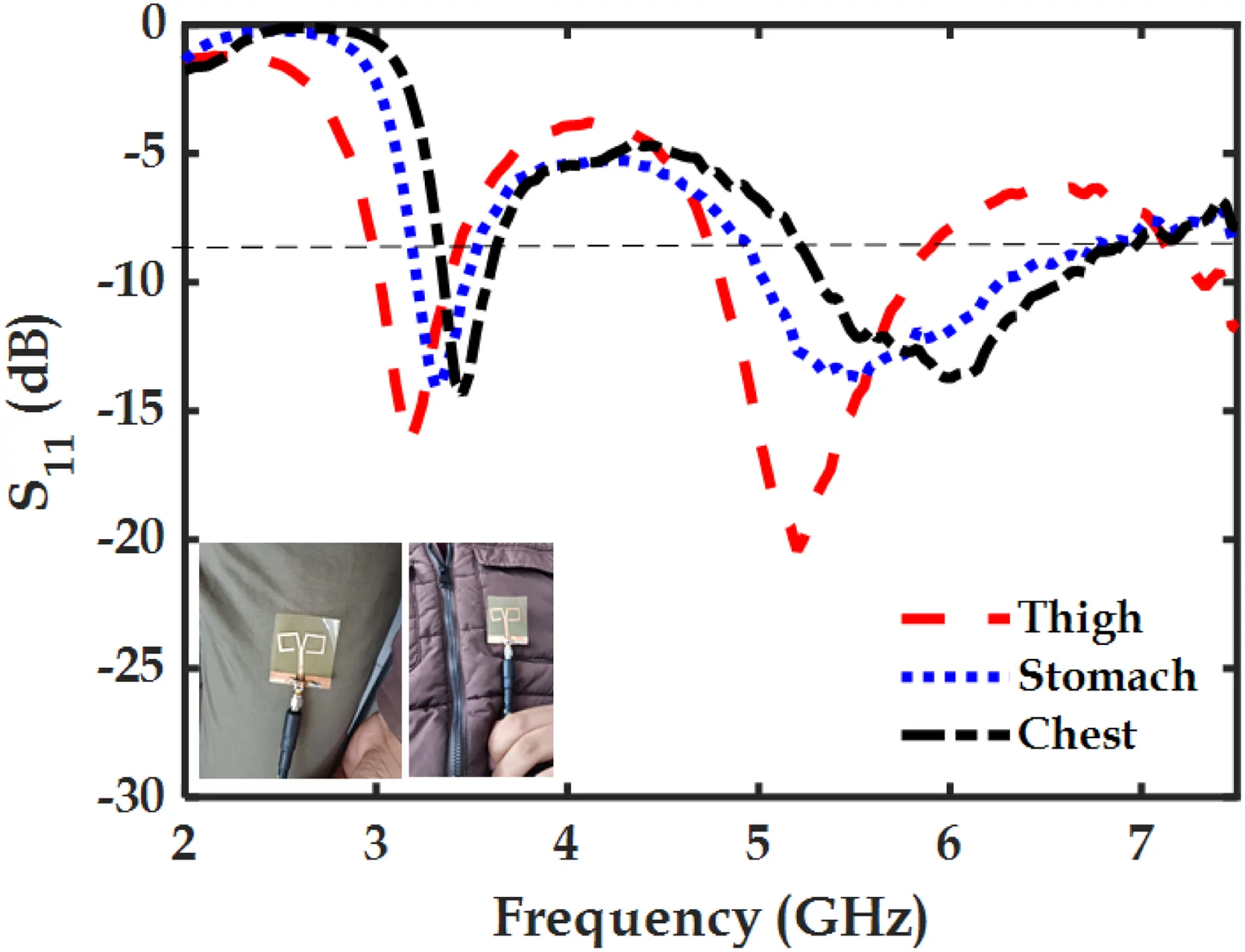
Measured reflection coefficient (|*S*_11_|) of the proposed antenna under on-body conditions at different locations. The inset shows the experimental setup.

In wearable antenna applications, the close proximity of the human body can significantly affect antenna performance due to the lossy and dispersive nature of biological tissues. Therefore, evaluating the antenna behavior under on-body conditions is essential to ensure reliable operation in practical scenarios. To investigate this effect, the proposed antenna was experimentally characterized when placed on different parts of the human body, including the thigh, abdomen, and chest. These locations were selected to represent typical wearable configurations with varying curvature and tissue composition. The reflection coefficient (|*S*_11_|) was measured using a vector network analyzer under realistic placement conditions, as illustrated in Fig. 13. The measured results indicate that the antenna maintains acceptable impedance matching (|*S*_11_| ≤ −10 dB) across the targeted frequency bands for all tested positions. However, slight shifts in the resonance frequencies are observed depending on the placement location. These variations are mainly attributed to differences in tissue composition, dielectric properties, thickness, and local curvature, which modify the effective permittivity surrounding the antenna and consequently affect its input impedance. In the higher frequency band, the antenna placed on the thigh exhibits improved impedance matching, with resonance levels approaching −20 dB around 5–5.5 GHz. In contrast, the abdomen and chest placements show similar responses, with minor frequency detuning and slightly reduced resonance depth. In the lower frequency band (3–4 GHz), all positions demonstrate comparable behavior, although small frequency deviations are observed due to the lossy nature of human tissues. These results confirm that the proposed antenna exhibits stable electromagnetic performance under on-body conditions, with limited sensitivity to variations in placement. This robustness highlights the suitability of the PVDF-based composite antenna for wearable and body-centric wireless communication applications.

### 3.4 Simulated radiation characteristics

**Fig. 14:**
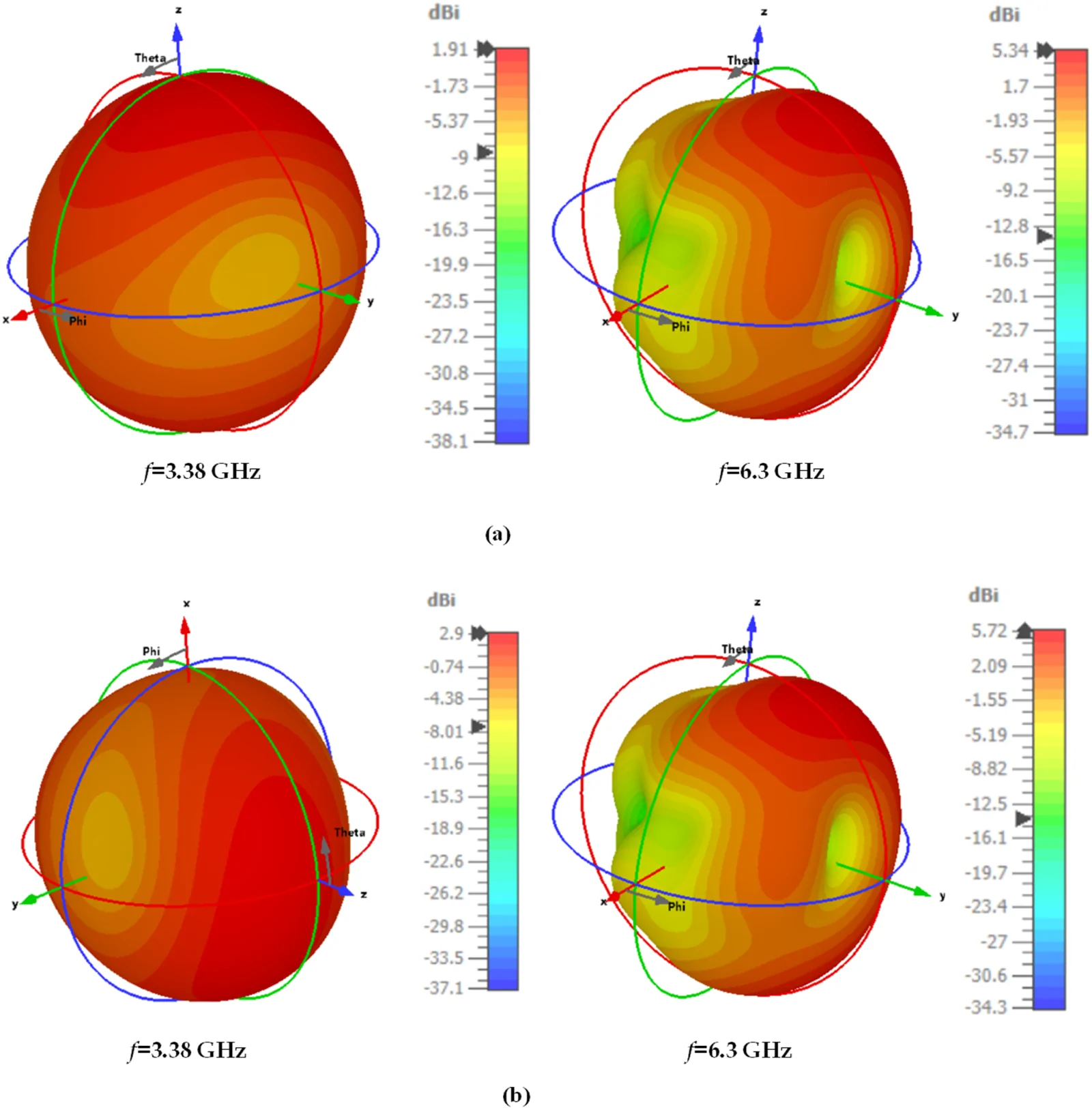
Simulated three-dimensional radiation characteristics of the proposed antenna: (a) directivity and (b) gain at 3.39 GHz and 6.30 GHz.

**Fig. 15:**
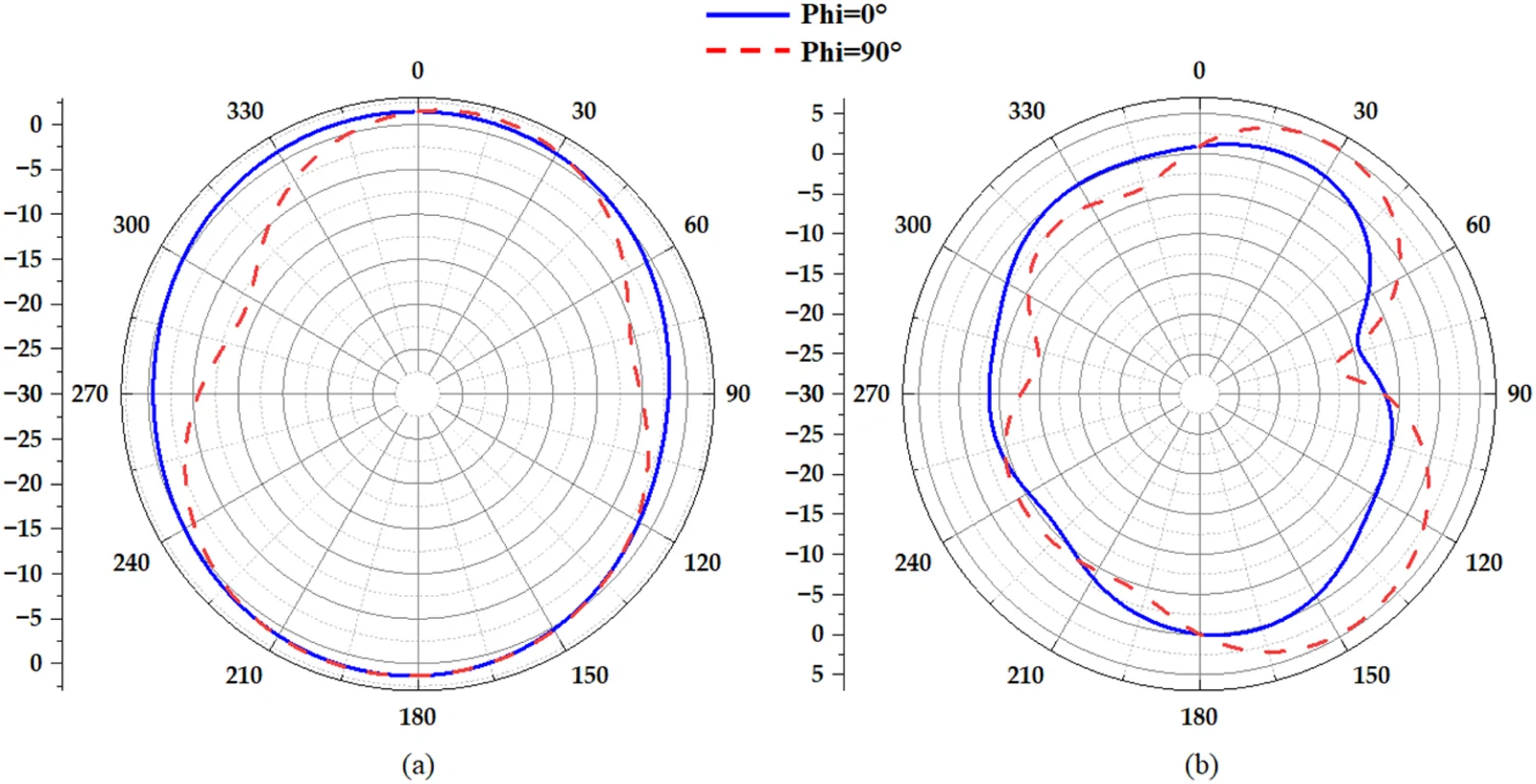
Simulated two-dimensional radiation patterns of the proposed antenna: (a) 3.39 GHz and (b) 6.30 GHz, for *φ* = 0° and *φ* = 90° planes.

The simulated three-dimensional radiation patterns of the antenna fabricated on the PVDF/3Ni–P substrate are presented in Fig. 14 for the resonant frequencies 3.39 GHz and 6.30 GHz. Fig. 14(a) illustrates the corresponding 3D directivity patterns, while Fig. 14(b) shows the associated gain distributions. At 3.39 GHz, the antenna exhibits a stable radiation pattern with the main lobe oriented in the broadside direction relative to the radiating structure. A similar radiation behavior is observed at 6.30 GHz, although slight variations in the radiation lobe shape occur due to the excitation of the higher-order resonant mode. Despite these variations, the antenna maintains effective directional radiation in the upper frequency band. The gain distributions presented in Fig. 14(b) further confirm that stable radiation efficiency is maintained at both resonant frequencies. The close correspondence between the gain and directivity characteristics indicates low radiation losses and efficient electromagnetic power conversion across the antenna structure. Finally, Fig. 15 presents the simulated two-dimensional radiation patterns of the CPW-fed antenna implemented on the PVDF/3Ni–P substrate at 3.38 GHz and 6.30 GHz. The results show that the antenna preserves stable radiation behavior in both operating bands, with quasi-omnidirectional characteristics in the *φ* = 0° plane and bidirectional radiation in the *φ* = 90° plane, confirming the suitability of the proposed antenna for dual-band wireless communication systems, radiation characteristics are obtained from full-wave simulations due to measurement limitations.

### 3.5 SAR evaluation

**Fig. 16:**
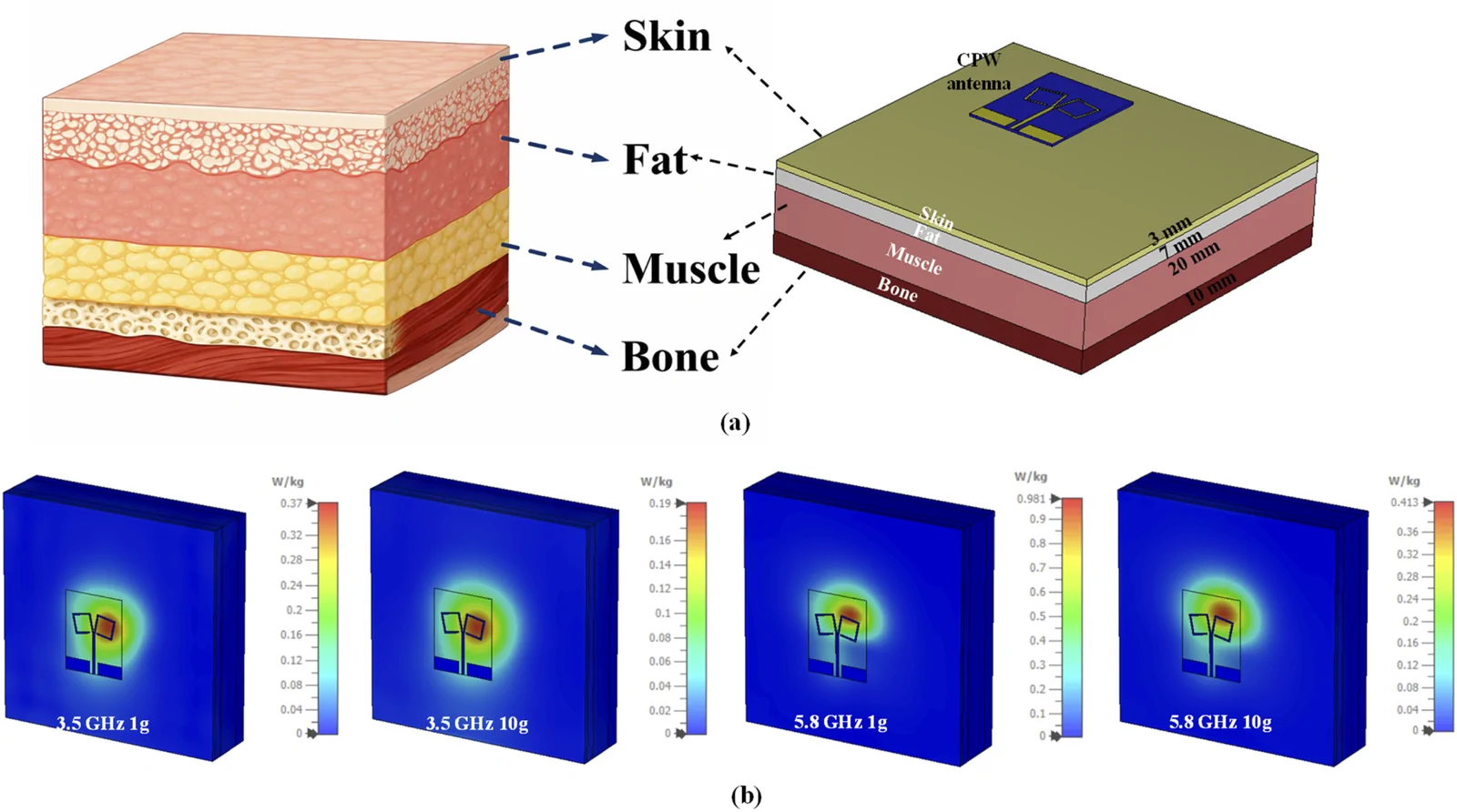
(a) Configuration of the proposed antenna positioned above the multilayer human tissue model. (b) Simulated specific absorption rate (SAR) distribution at 3.5 GHz and 5.8 GHz.

In wearable antenna applications, the close proximity of radiating elements to the human body necessitates careful evaluation of electromagnetic exposure to ensure user safety. The specific absorption rate (SAR) is a key parameter used to quantify the amount of electromagnetic energy absorbed by biological tissues. To assess the safety performance of the proposed antenna, SAR simulations were carried out using a multilayer human tissue model implemented in CST Microwave Studio. The model consists of a cuboid structure with dimensions of 100 × 100 × 40 mm^3^ and includes four biological layers representing skin, fat, muscle, and bone, as illustrated in Fig. 16(a). The electromagnetic properties assigned to each tissue layer were taken from standard literature.^2^ The antenna was positioned at a separation distance of 10 mm above the tissue surface, corresponding to a typical wearable configuration. The simulations were performed in accordance with IEEE/IEC 62704-1 guidelines, assuming an input power of 100 mW. According to international safety standards, the SAR must remain below 1.6 W kg^−1^ averaged over 1 g of tissue (U.S. regulation) and 2 W kg^−1^ averaged over 10 g of tissue (European regulation). The SAR is defined as:^40^1
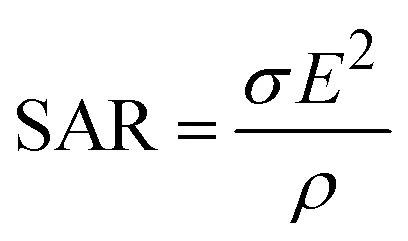
where *σ* represents the electrical conductivity of the biological tissue, *E* denotes the local electric field magnitude, and *ρ* corresponds to the mass density of the tissue.

The simulated SAR results indicate that, at 3.5 GHz, the maximum SAR values are 0.37 W kg^−1^ averaged over 1 g of tissue and 0.19 W kg^−1^ averaged over 10 g. At 5.8 GHz, the peak SAR values increase to 0.981 W kg^−1^ (1 g) and 0.413 W kg^−1^ (10 g). These values remain well below the limits defined by both U.S. and European safety standards. The spatial distribution of SAR within the tissue model is illustrated in Fig. 16(b) for both operating frequencies. As expected, the highest SAR levels are concentrated in the skin layer closest to the antenna, with rapid attenuation observed in deeper tissues. These results confirm that the proposed antenna satisfies international safety requirements and can be safely used in wearable applications. A minimum separation distance of 10 mm is recommended to ensure continued compliance with electromagnetic exposure guidelines. The peak SAR values summarized in Table 3 are significantly below the international safety limits of 1.6 W kg^−1^ (1 g) and 2 W kg^−1^ (10 g). These results confirm the safe operation of the proposed antenna for wearable applications.

**Table 3:** Peak specific absorption rate (SAR) values of the proposed antenna at 3.5 GHz and 5.8 GHz compared with international safety limits

Unit (W kg^−1^)	Standard (max value)	3.5 GHz	5.8 GHz
1 g standard (US)	1.6	0.37	0.981
10 g average (EUR.)	2.0	0.19	0.413

## 4 Conclusion

In this work, a compact coplanar waveguide (CPW)-fed dual-band antenna based on a metamaterial-inspired configuration is proposed for sub-6 GHz applications. The antenna is implemented on electroactive polyvinylidene fluoride (PVDF) composite thin films incorporating metal phosphate fillers (Ni, Ag, and Co), which serve as flexible dielectric substrates. Prototype antennas fabricated on PVDF/3Ni–P and PVDF/3Co–Pn substrates are experimentally characterized using a vector network analyzer. The proposed design features a compact size of 40 × 52 mm^2^ (approximately 0.47*λ*_0_ × 0.61*λ*_0_ at 3.5 GHz). Dual-band operation is achieved at 3.5 GHz and 5.8 GHz, covering sub-6 GHz and industrial, scientific, and medical (ISM) bands. The measured impedance bandwidths extend from 3.17 to 3.50 GHz and from 5.62 to 7.16 GHz, showing good agreement with simulated results. The antenna achieves peak gains of 1.56 dBi and 5.32 dBi, with radiation efficiencies of 80% and 91% in the lower and upper bands, respectively. Bending analysis confirms stable electromagnetic performance under mechanical deformation, demonstrating the structural robustness of the proposed configuration. In addition, the maximum specific absorption rate (SAR) remains within international safety limits, ensuring safe operation in proximity to the human body. These results demonstrate the effectiveness of electroactive PVDF-based composites as multifunctional dielectric substrates for flexible dual-band antennas for 5G, IoT, and wireless body area network (WBAN) applications. Future work will investigate the integration of the intrinsic piezoelectric properties of these materials with RF antenna technology to develop hybrid wireless communication and energy-harvesting platforms, paving the way toward future self-powered wireless systems.

## Conflicts of interest

There are no conflicts to declare.

## Data Availability

The data that support the findings of this study are available from the corresponding author, Said DOUHI, upon reasonable request.
